# A ‘worthy disciple of Galen’, ‘ardent sportsman’ and ‘expert swordsman’: Henry Kipping (1726–1785) apothecary and surgeon at Brighton, England

**DOI:** 10.1177/09677720231190856

**Published:** 2023-07-27

**Authors:** Maxwell J Cooper, Jason Heath

**Affiliations:** 1 Department of Primary Care and Public Health, 12190 Brighton and Sussex Medical School, Brighton, UK

**Keywords:** Brighton, England, apothecary, sussex, horseracing, hunting, resuscitation, duel

## Abstract

Henry Kipping (1726–1785) was an apothecary and surgeon in Brighton, England. Here we present a series of contemporary references to Kipping from newspaper, book, archive and web-based resources. Some relate to his medical practice (resuscitating a ‘drowned’ elderly physician and a fisherman, bleeding a member of parliament who had fallen from his horse and praising a nostrum for the ‘gravel and stone’). Social references include a duel with an army officer whose sword Kipping confiscated. Kipping appears to have been popular, connected with members of Brighton’s high society and passionate about traditional past times, e.g. swordsmanship, horse riding and hunting on the Sussex downs. Indeed, Kipping’s horse ran in the earliest known horse race in Brighton (1770). He was consulted by notable local residents including the Thrale family of Brighton and Lady Wilhelmina Shelley (the latter evidenced by Kipping partaking in her funeral procession in 1772). Kipping lived and practised at 28 West street, a road most famous for its (now lost) George Inn where King Charles II stayed just prior to his escape to Normandy. Kipping comes across as a colourful and eccentric clinician.

## Background

In the first half of the eighteenth century medical services in Brighton, England, were provided by local apothecaries. This is evident in the burial record of St Nicholas’ church, Brighton, which reveals a ‘Thomas Barton apothecary’ buried there on the 25th February 1743. More formal treatment may have taken the form of visiting physicians and surgeons from nearby Lewes, the county town of Sussex. In the middle of the eighteenth century, the Lewes physician Richard Russell (1687–1759) established himself in Brighton and built a successful practice there largely based upon seawater therapy. Seawater, mineral water or steam therapy was continued in Brighton by other notable doctors for example Anthony Relhan (c.1715–1776), John Awsiter (1732-1801), Robert Henderson (d1808)^1^ and Sake Dean Mahomed (1759–1851). In the mid-eighteenth century, other practitioners recorded at Brighton included Dr Schomberg (son of Dr Schomberg of London)^2^ and Dr William Brodum.^3^

Local practitioners later in the century are listed under ‘Brighthelmstone’ (the original name for Brighton) in Bailey's British Directory for 1784 (see Table 1)^4^ and the 1791 Universal dictionary (see Table 2).^5^ No earlier directory containing Brighton medical practitioners appears to have survived. It is of note that certain doctors provided services in Brighton only for ‘the summer season’ or ‘bathing season’. This highlights the status of the town as an expanding resort for royalty, the rich, and the infirm. Seasonal doctors included: Dr Poole of Lewes (in 1760),^6^ Dr Chitteck,^4^ Richard Kentish (in 1787)^7^ and Sir Lucas Pepys (1742–1830).^4^ Dr Chitteck appears to be the same doctor of Bath, Somerset, whose nostrum for bladder stones was later reported to be a but an alkali soap. Dr Kentish sought employment in Brighton, wrote the preface of his book (on seawater therapy) whilst in the town^8^ and may have practised there later on a more permanent basis. A number of less well known doctors are included in Table 1, amongst whom is the topic of this paper namely Henry Kipping senior (1726–1785).

**Table 1.** Brighton doctors recorded in Bailey's British Directory for 1784.

Chitteck physician for the summer season
Kipping, Henry surgeon and apothecary
Lowdell, George and Isaac surgeons and apothecaries
Pepys, Sir Lucas ‘physician for the summer season’
Tillstone, Richard surgeon and apothecary

**Table 2.** Brighton doctors recorded in the Universal dictionary of 1791.

Michael Cobby, surgeon and apothecary
John Cocks, surgeon and apothecary
J Collard, chymist and druggist
John Cristall, surgeon and apothecary
John Hall, surgeon and apothecary
John Hargraves, surgeon and apothecary
R Hendersen M. D. – Kipping, Surgeons, &c
Pankhurst, Surgeons, &c. Richard Tillstone
Surgeon, apothecary & man-midwife

## Introduction

This paper presents references to the life of Henry Kipping. These relate to diverse events and are presented here as individual accounts in chronological order (where possible). One undated description offers insight into Kipping as a person:

The Doctor, though a worthy disciple of Galen, was an ardent sportsman and an expert swordsman. Fighting once an impromptu duel in West-street, where he resided, with an Officer who had insulted him, he took in the encounter the Officer's sword; keeping it for over a week, much to the latter's chagrin^9^

The reference to ‘sportsman’ likely relates to country activities, in particular fox hunting, horse riding, etc. This rural interpretation of the word is supported by further accounts presented later below.

## West street, Brighton

Kipping lived at 28 West street, Brighton, and an extant lease document from 1830 suggests that this was on the ‘west side of west street.^10^ One source states that in 1929, 28 West street was sold for ‘road improvement’ and changed to number 26.^11^ Today it is not possible to identify the precise location of Kipping's house. The house must have also functioned as his ‘surgery’, i.e., his domestic home and clinical premises where consultations and operations were undertaken. The property was later used by his son Henry Kipping junior (died 1804), also a surgeon. In 1800 the house was the surgery of ‘Kipping, Pankhurst, and Barrett’. West street is also noted in 1803 as the location of the ‘great house’ of the army physician Dr Robert Henderson (died 1808).^12,13^

**Figure 1.**
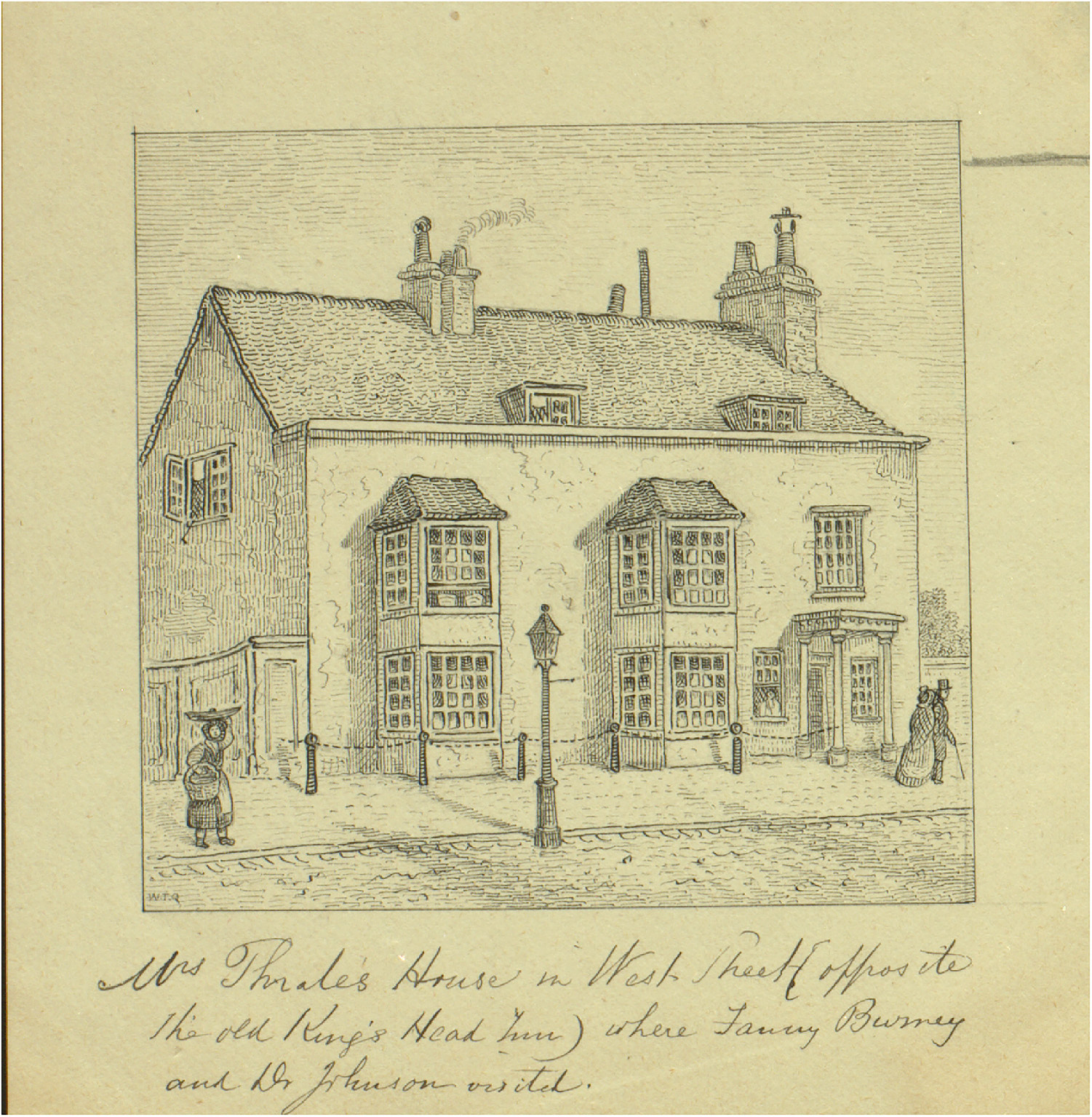
Mrs Thrale's house on West street, Brighton. Image by William Thomas Quartermain. The only surviving part of this home is the first (i.e. on the left) of the five posts. This is visible today (see image 2). Image courtesy of the Royal Pavilion & Museums, Brighton & Hove.

**Figure 2.**
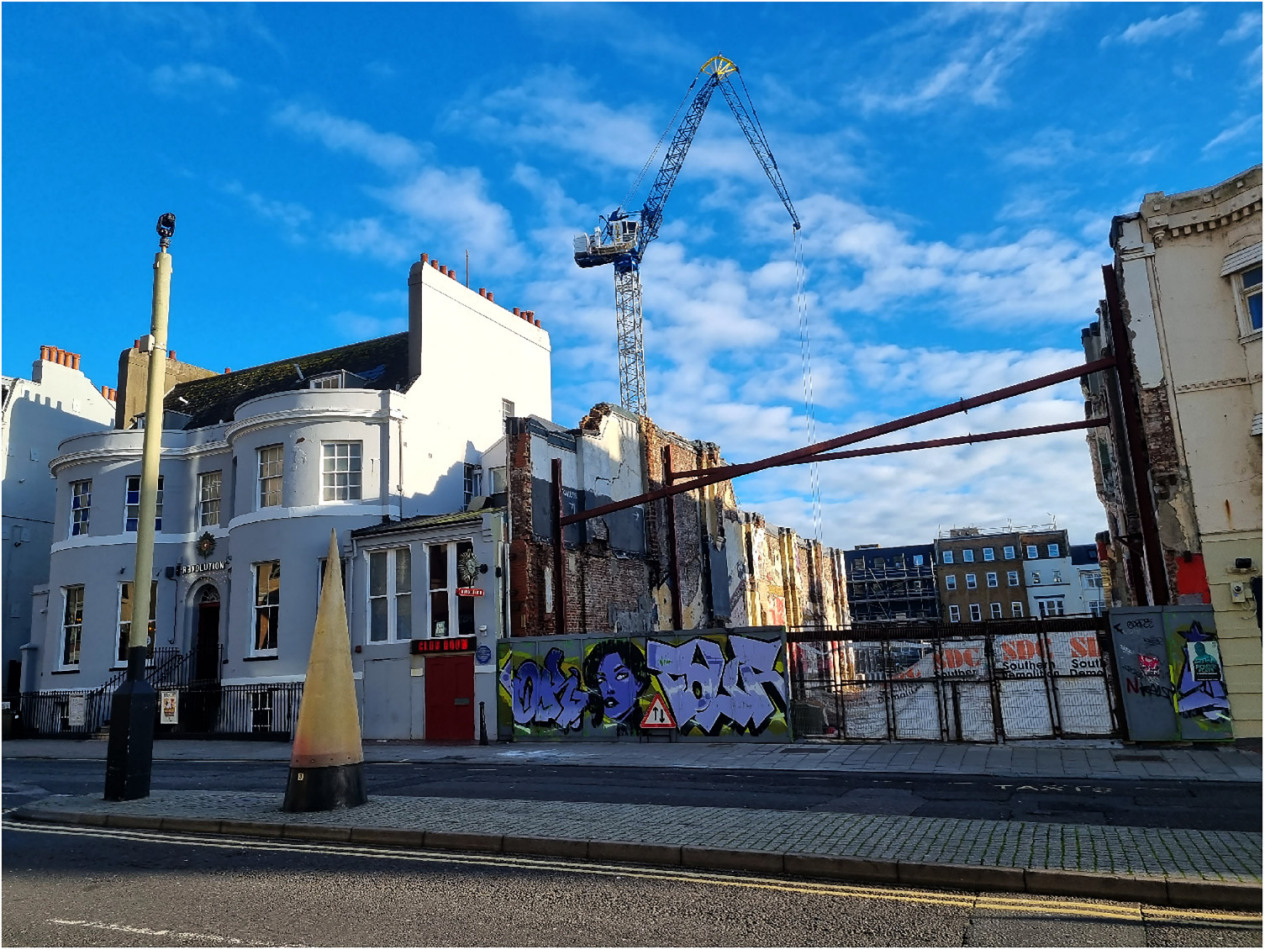
The site of the Thrale's house at the South end of West Street, Brighton. The only remnant of the building is the small black post just visible to the left (beneath blue plaque on house). This was probably used for tethering horses. Image taken by MJF Cooper on 6 December 2022.

In the eighteenth century West street was a prosperous part of the town and one remembered for visits by notable figures. Other residents included the Thrale family whose house stood at the south (nearer the sea) end of West Street next to the town customs house (see Figures 1 and 2).^14^ Visitors to the Thrales included the writers Samuel Johnson (1709–1784) and Fanny Burney (1752–1840).

No image or description of Kipping's house exists. One 1740 account of a London apothecary shop front may, in lieu, offer insight: That apothecary shop was “well stored with Medicine … [and] it is ornamented by the Apothecary … by a Museum or Cabinet of various Curiosities; another with a beautiful collection of the *Materia Medica*, and a large handsome fram'd skelleton [sic] placed over the same, with other Decorations, or Ornaments"^15^

**Figure 3.**
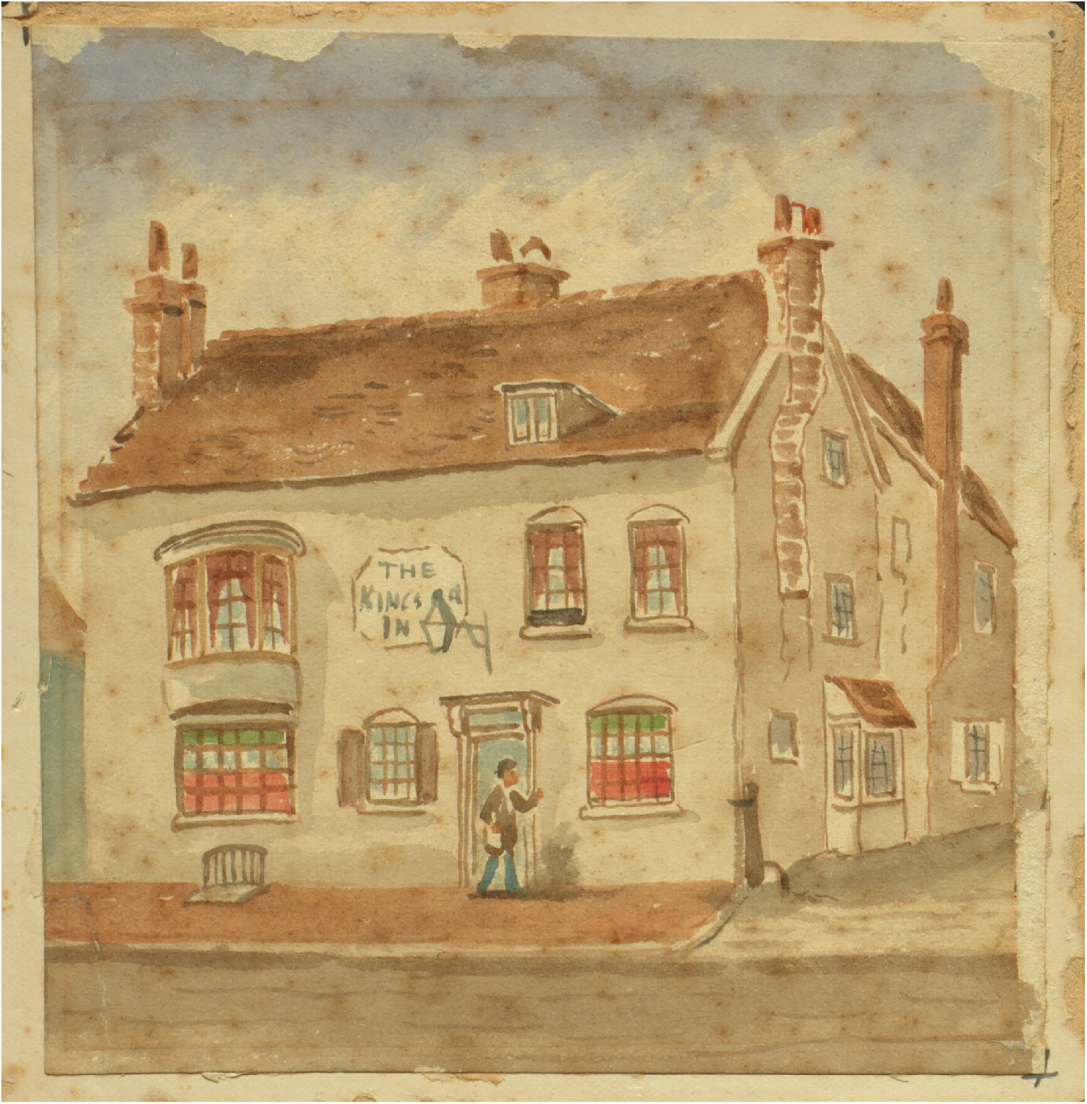
The King's Head, West Street, by William Thomas Quartermain. Formerly known as the George Inn, it is situated close to the site of Kipping's house. King Charles II stayed here the night before his escape to France on 15 October 1651. For many years after an oak branch was hung annually on the building to commemorate the event. Charles’ escape is remembered today in Brighton's annual Royal Escape yacht race. Image courtesy of the Royal Pavilion & Museums, Brighton & Hove.

Further insight into the character of West street might be gained from contemporary buildings. Most notable on the west side of West street stood the George Inn (see Figure 3), later the King's Head. Writing from Brighton in 1778, Fanny Burney notes the following history:

Mrs Thrale's house [i.e., adjacent to Kipping's house, see Figures 1 and 2] is at the Court end of the town, exactly opposite to the King's Head, where Charles II stayed just before his escape to France.^16^

It was here that King Charles the first stayed on the 14 October 1651, the night before his escape to Normandy. It is recorded that ‘for many years after a branch of oak was hung out through a top window each year to celebrate his stay’ (presumably upon the anniversary).^17^

## Early references to the Kipping family and household (pre-1770)

Kipping was associated with Brighton as early as 1756. The parish records of St Nicholas’ church, Brighton note the following under burials:

December 6 1756: Elisabeth wife of Mr Henry Kipping

July 6 1757: James Day servant to Mr Kipping, small pox

April 17 1760: Susanna the wife of Mr Henry Kipping

September 30 1760: John son of Mr Henry Kipping & his late wife

In 1768 ‘Henry Kipping’ was appointed a trustee of Grimmet's educational charity, Brighton.^18^ The same source indicates that its moneys came from ‘Old South Sea annuities’.^17^ It is almost certain that his medical training followed a traditional apprenticeship and one source states that this was under Josiah Higden, a London apothecary.^19^ Such a conclusion would indicate a formation with oversight from the Worshipful Society of Apothecaries. The association with Josiah Higden may be uncertain given that records (see later) reveal two contemporary surgeons named Henry Kipping and associated with Sussex/Kent.

## A passion for horses (1770)

Henry Kipping is recorded as being a racehorse owner. This account is the first reference to horse racing in Brighton:

The earliest horse racing at Brighton we know of was a four-mile heat on the Downs, on March 5th, 1770, between the horses of Mr Shergold (of ‘The Castle’) and of Dr Kipping, for 10 guineas a side, the stakes being won by the Doctor's horse.^9^

The contemporary description of this race is as follows and suggests that a course was already established:

LEWES March 5 Saturday last one four-mile heat was run on our course, by a Horse belonging to Mr Samuel Shergold, carrying twelve stone, and a Mare, the property of Dr Kipping, both of Brighthelmston, carrying ten stone, for ten guineas a-side, which was won by the latter. Mr Shergold's rider was a stranger to the course, and rode out of it some distance, which some think was the cause of his losing.^20^

The sums involved above point to significant financial stakes in horseracing, presumably for owners as well as gamblers. This – and the location of Mr Kipping's house – suggest that he was financially well off and had sufficient free time to pursue wider interests.

## Treating the casualty of a fall (1771)

A newspaper account from 1771 describes Kipping using bloodletting as a treatment:

Monday night last as James Hunsted, who was servant to the late Mrs. Bernard of this town, was walking along the West street in Brighthelmston, it being dark, he fell down an area belonging to the house of Sir John Shelley, Bart. whereby he cut and bruised himself in so terrible a manner, that he was taken up speechless, and without signs of life; but being carried to Mr Kippin's [sic], apothecary and surgeon, (who lived just by) he was blooded, and brought to himself, after which the above gentleman carefully dressed his wounds, and administered such other effectual remedies, that the next day afforded the patient the most promising hopes of a speedy recovery. A little boy who stood not far from the place, luckily saw him fall, and went and got immediate assistance, which in all probability, preserved his life, as had he laid long, ‘tis thought he must have been choaked [sic] with blood, he being greatly [?] cut above the face.^21^

## Joining the funeral procession of a noblewoman (1772)

**Figure 4.**
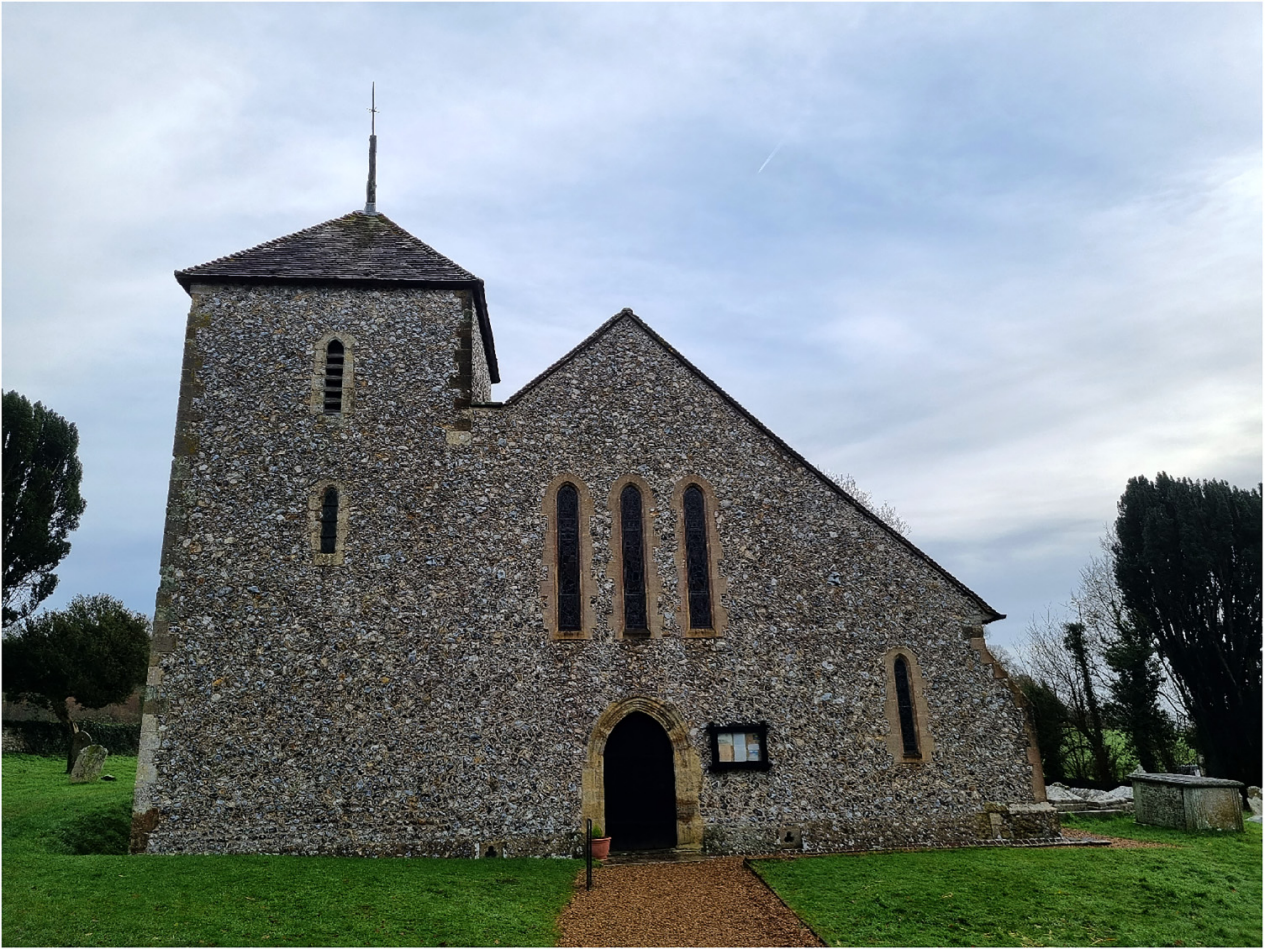
St Mary the Virgin, Clapham, Sussex. On the 2nd March 1772, Henry Kipping joined the funeral procession of Lady Wilhelmina Shelley. On this morning the church was closed. Image taken on 24 December 2022 by MJF Cooper.

On 2 March 1772, Lady Wilhelmina Shelley died presumably as a result of obstetric complications. Kipping is recorded as part of her funeral procession at St Mary the Virgin (see Figure 4), Clapham, West Sussex on 29 March 1772:

The Corpse of the late Lady Shelley being yesterday brought from London to Horsham & was this day brought from thence to Steyning about Eleven o’clock attended by Mrs Windsor the Housekeeper & Mrs Coward my Ladys woman Mr Cook the Cook & Thomas Whitington my Ladys footmann where Mr Henry Shelley Doct. Kipping of Brighton & Capt. Henry Bishop and all Sir John Shelleys Tenants & a great number of his Tradesmen from Arundel joyned the Corpse & attended to Clapham Church where it was interr’d near the Communion Table within the Rails of ye Chancel about four o’cloc^22^

## A missing dog (1774)

Mr Kipping is mentioned in an advertisement in 1774 for a lost dog:

STOLEN or Stray’d from the Town of Brighthelmston, A young POINTING DOG; About twelve Months old, unbroke, of a light Liver Colour and White, answers to the Name of DON, had round his Neck when lost, a Collar, with the Name of JOHN NORRIS, Esq; MOUNT STREET, GROSVENOR SQUARE. Whoever will bring him to Mr Kipping, Surgeon, in Brighthelmston, shall receive Half a Guinea Reward. No greater Reward will be offered.^23^

## Resuscitating a ‘drowned’ physician (1774)

In 1774 Kipping wrote to the Royal Humane Society about a successful case of resuscitation:

EXTRAORDINARY INSTANCE OF RESUSCITATION BY MR. KIPPING.

A.B. a Physician, upwards of 70 years of age, was underwater for about *7 minutes*. As I had the distance of *4 miles* to ride, it must take *30 minutes*, or more, from the messenger's setting out and my arrival at the house. – His face and lips were livid, body universally cold, and other strong appearance of dissolution. – The usual means were employed; and, in about *15 minutes,* the circulation returned.

In about *20 minutes* more, evident signs of life appeared, by a gradual return of warmth, &c. and soon afterwards my patient became sensible, and expressed a strange surprise at what the people about him were doing. In about an *hour*, I got down some warm sack-whey, and still employed the resuscitative process.

During the whole day, the sea water, which had been received originally into the stomach, was discharged in great quantities by the renal glands.

Brighton, July 29. H. KIPPING^24^

The account above shows that Kipping was familiar enough with the anatomy and physiology of the body to know that water was excreted by the kidneys. The event is described thus in the Sussex Advertiser:

On Monday last a Physician from Brighthelmston was drowned as he was bathing in the sea at the Salt dean gap. He was soon after taken up and carried to Rottingdean, when Dr Kipping, of Brighthelmston, was immediately sent for, who on his arrival, proceeded to use the methods prescribed by the Medical Society for the recovery of drowned persons, which, in about three quarters of an hour, had the desired effect, and the Gentleman's health we hear, is now perfectly restored.^25^

This account is interesting as it shows a clear intention to conceal the patient's identity. This is unusual for this newspaper; as is evident in other accounts presented below, it routinely named patients in its sections on news. Despite searching, we have failed to identify the physician in question although we note the uncertainty over the date of birth of Anthony Relhan and that his initials resemble ‘AB’. It is most likely, however, that the physician was visiting from elsewhere or retired to Brighton, perhaps on grounds of poor health. Entering the water would align closely with someone seeking the health benefits of the town.

The description of resuscitation aligns broadly with other accounts of near-drowning in Brighton.^13^ It is of note that the actual method of resuscitation employed is never mentioned. In this case, the recovery appears to have taken up to an hour and his recovery may, therefore, have resulted from warming the body and clearance of ingested saltwater rather than the resuscitation techniques employed. Modern day accounts of recovery from periods of prolonged immersion with hypothermia might support this.

## Treating Ralph Thrale, a child with a complex headache (1775)

A further reference to Kipping comes in 1775 relating to the treatment of Ralph Thrale (1773–1775), whose family also lived on West street. Thrale had been suffering from a condition that was likely to be congenital hydrocephalus.^26^ Kipping, it appears, had recognised the condition instantly because he had ‘lost one child by this Disorder and [had] one alive who [was] an Ideot [sic]’.^26^ This deeply personal experience may have made Kipping reluctant to intervene in Ralph's case, instead deferring to Sir Lucas Pepys. Mrs Thrale notes:

[Ralph was] more heavy more lethargick & insensible than ever I had known him at home: Old Nurse talked of Teeth again, but I soon saw Teeth had a small share in his Complaint & apply'd to Kipping the Apothecary of the Place, who immediately said his Brain ws oppress’d & beg'd me to consult Dr Pepys^26^

Mrs Thrale's own opinion concurred with Kipping's: ‘[Ralph's] fits of Rage – proceeding from Pain I guess – just as Lucy & Miss Anna had – Kipping says the Brain is oppressed of which I have no doubt. What shall I do? What can I do?’^26^

Another letter from Mrs Thrale (this time to Dr Samuel Johnson) states the following: ‘[Ralph's] Muscular Flesh however seemed rather to increase than diminish, & as Reason appeared to be in greater Danger than Life I left him on the 8.th under the Care of Kipping[,] Pepys & old Nurse & returned to Streatham’^26^

Mary Hyde later describes the events after Ralph Thrale's death in 1775 thus:

When Mrs. Thrale made her lonely trip back to Brighton on 13 July, Kipping and Scrase gave what consolation they could, and the latter made the burial arrangements. In St Nicholas's Church, at the west end of the nave, is a monument stone to Ralph Thrale.^26^

## Treating W Gerald Hamilton MP who fell while riding his horse (1775)

Kipping senior is also mentioned in relation to resuscitating the Right Honourable W. Gerald Hamilton MP: ‘Mr Hamilton (once reputed to be the author of Junius) was among the earliest visitors to Brighton. He nearly lost his life on the Downs in 1775: From his horse “cannoning” against Sir Ferdinand Poole's, whilst hunting. He was rendered insensible; but was eventually restored by Dr Kipping, who happened to be on the field’^9^

On 27 November 1775, Kipping is again recorded undertaking bloodletting. This account suggests that Kipping was present on the hunt, one presumably undertaken with hounds:

On Monday last, as Sir Ferdinando Poole, Bart. And – Hamilton, Esq. were hunting together on the Downs, near Brighthelmston, they rode against each other with such force, that both their horses fell, whereby Mr Hamilton was so violently stunned, that he was taken up to all the appearances dead; but Mr Kipping, of Brighthelmston, luckily being present, he let him blood, which soon relieved him, and he is now perfectly recovered. Sir Ferdinando Poole received very little hurt.^27^

## Resuscitating a fisherman (1776)

On 6 May 1776 Kipping is noted in conjunction with Mr Lowdell surgeon of Brighton (see Table 1):

On Friday last as John and henry Baker, Ferryman [sic] at Brighthelmston, were bringing a Fisherman on shore, their boat overset, by which accident the two former (brothers) were drowned, but being soon after found and taken up, they were carried to different public houses, where the methods prescribed by the Society for the recovery of drowned persons, being used by Dr Kipping and Mr Loudle [sic], in about half an hour they were both brought to shew signs of life and are now perfectly recovered^28^

## A letter on resuscitation by Mr Kipping (1777)

The only piece of writing from Kipping's pen is a letter published by the Royal Humane Society. It describes the two cases presented above:

CASE CLXVII

DR. KIPPING TO DR HAWES

Brighton, May 6. 1777

SIR, I have received the favour of your letter and Reports, for which I heartily thank you, and at the same time I acknowledge my thankfulness for the kind attention you have been pleased to pay to the case which I sent you last summer.

And as I am sure it will manifestly confirm the great utility of so humane and noble an Institution, I have now the satisfaction and pleasure to remit to you TWO INSTANCES, which have within these few days occurred, sufficient to convince the world how vastly superior the methods recommended by your Humane and the Philanthropic Society, are to any of the resuscitative plans formerly employed for restoring drowned or otherwise suffocated persons.

May 2. TWO FISHERMEN going off in a small boat to take some mackarel [sic], from a boat opposite to this town, in their return to shore, by a sudden squall of wind, and a great sea, they were overset, and the men unhappily caught under the boat, and in so dreadful a state they continued for a *quarter of an hour*, before any body could afford them any assistance, from the great roughness of the sea.

I attended at the Receiving-house as soon as they were brought to shore, and one of the men was so far gone as to afford no hopes of recovery. But, by immediately pursing with zeal and diligence the methods recommended by the truly HUMANE SOCIETY, in about *a quarter of an hour* I began to perceive a manifest glow and warmth *about the praecordia*, and a gradual diffusion of this essential principle of vitality flow.

By persevering in the various means of Resuscitation, he became more and more sensible, and is now perfectly restored.

THE OTHER MAN is likewise well, but his symptoms were much more favourable, owing to his being able naturally, and immediately, to discharge a large quantity of sea-water from his stomach. I most sincerely wish success to your valuable philanthropic Institution.

HENRY KIPPING^29^

## Treating the ‘stone and gravel’ (1777/8)

Kipping is mentioned in 1778 in a review of a medical book written by Nathaniel Hulme M.D. entitled ‘A safe and easy Remedy proposed for the Relief of the Stone and Gravel, the Scurvy, gout, &c. and for the Destruction of Worms in the Human Body’.^30^ This review reads as follows: ‘THE remedy here advised by Dr Hulme is the same as that which he recommended last year, in the cure of the stone. It consists of a solution of salt of tartar in water, so that the mixture of these medicines may produced fixed air in the stomach. The efficacy of this method of cure, Dr Hulme now confirms by four other cases which have fallen under his own observation, as well as by a case transmitted by Dr Hossack, at Colchester, and one of the same nature by Mr Kipping, apothecary at Bighthelmstone’.^30^ Hulme's book goes onto describe Kipping's account:

By a Letter from Mr Henry Kipping, Apothecary at Brighthelmstone, dated Dec.2, 1777, I am informed that ELIZABETH CROUCH, an inhabitant of that town, has found great relief, in a nephritick complaint from the use of the remedy here recommended. That since taking the medicine, which she has done regularly for five months, she has had only one fit, which continued but a short time; and that she has voided, at different periods, many small stones, one of which was considerably larger than the rest.^31^

## An account of Mr Kipping's character (1779)

In 1779 the English novelist and author Fanny Burney (1752–1840) recorded the following encounter with Kipping:

The morning after our arrival, our first visit was from Mr Kipping, the apothecary, a character so curious that Foote designed him for his next piece, before he knew he had already written his last. He is a prating [sic] good humoured, old gossip, who runs on in as incoherent and unconnected a style of discourse as Rose Fuller, though not so tonish^32^

## Advising on Mr Thrale’s illness (1779)

On 1 December 1779, the following is included among the diary records of Mrs Thrale:

On Monday last the 22 of November he complained of the headache, eat [sic] no Dinner & looked most dreadfully – [Sir Lucas] Pepys was gone to London, so I had nobody to consult but Kipping the Apothecary who modestly & wisely would do nothing but advise me to make haste with my Master to Stretham. We accordingly set out yesterday Tuesday 23: – it was our intention before he was ill to leave Sussex that day – and the Weather was very Cold. Mr Thrale said his head ached, but when we got to Cookfield he had such a shivering & Torpor came on as shocked me, & set poor Miss Burney o’ [sic] crying – his Wits were quite unsettled, & his Articulation almost wholly lost; Dinner revived him a little, but the Evening & the Night he was comatose, & I called in Heberden in the Morning, he order Cupping which restored him so far that we are now just as we were before his last Attack^33^

## A burglary at home (1783)

On 28 July 1783 the following account is given of a burglary at Mr Kipping's house. This lengthy newspiece is abbreviated here in order to include both references to Kipping, a portion that also offers insight into the nature of a burglary at this time:

Early on Thursday morning last, the house of Mr Kipping, surgeon and apothecary, in West street, Brighthelmston, was broke into and robbed of plate to the value of near [unclear, looks like ‘3ol’] by three persons, names Robert Parsons, Thomas Jones, and Abraham Abrahams alias Johnston, who were Friday morning last apprehended and secured at Uckfield, by Mr Edward Blackman, master of the Swan inn, in Southover, near this town, at whole house the above villains lodged the day and night after they had committed the robbery; and through whose vigilance and activity a box, containing the stolen articles and a variety of pick-lock-keys, was traced out in the possession of one Dale, (a gardener to a gentleman in Southover) and related to Parsons) to whose care the prisoners had committed it to be forwarded the next morning by the stage coach to Mrs. Nicholls, No. 21, in Hatfield-street, Goswell street, London, as directed. The box was detained and produced on the examination of the prisoners before Henry Shelley, Esq, where they were ready to acknowledge said box, with its contents, but denied the robbery, by roundly asserting that they had found the plate and picklock keys, tied up in a handkerchief, on the road between Shoreham and Brighthelmston, on the morning the robbery was committed. They agreed tolerably well in their story so far as it related to the bare finding, but in most other respects it was various and contradictory. Mr Kipping having sworn to the plate, and Dale to the reception of the box from the prisoners, (unknowing its contents) they were all three committed to the house of correction for further examination.^34^

The passage above goes on to mention that the three men were also wanted for a burglary in Wapping. The following month the paper suggests that two of the culprits were destined for capital punishment:

Last Saturday the assizes for this county ended, at which eleven prisoners were tried, five of whom were capitally convicted, and received sentence of death, viz…… Thomas Jones, and Robert Parsons, for a burglary, in the house of Mr Kipping, at Brighthelmston, on the 26^th^ of July last^35^

According to a modern reference to the incident: ‘The press coverage suggests that Mr Kipping, a surgeon who lived in Brighton, was assaulted during the theft and this would have decided the fate of the thieves’.^36^

## Mr Kipping’s death and will (1785)

On 22 April 1785 a letter to Hester Maria Thrale written at Venice contains the following entry:

Sophy tells me poor Kipping is sick; God bless my Soul Ma’am, why now these Things will sometimes – but if the Dr would come down indeed, but then, the Time o’ Year & that – & besides now you know Ma’am the Dr God bless him – but he is just the same as ever for the matter of that. &c

Poor Kip! I hope he will recover^37^

On the 4 June 1785 a letter to Hester Maria Thrale written at Florence states:

give a kind Word from me to Mr Kipping. Is poor Presto alive still? I have not seen one pretty dog in my Travels, the Race of Dutch Mastiffs or Pugs extinct in England or nearly so, are the fashionable Favourites of the Ladies at Padua^36^

The Sussex advertiser records for 1785 could not be accessed for this study and an earlier search did not note any mention of Kipping's death.^38^ Kipping's death could not be identified in transcripts of the burial records of St Nicholas’ church, Brighton. In 1786 his creditors were sought in the local newspaper.^39^

His will is handwritten and extremely challenging to decipher.^40^ Here Kipping is described as ‘of Brighthelmstone in the county of Sussex Surgeon’. It is not clear which year he signed the will, which states: ‘the sixth of January in the year of 177[unclear]’. Henry Kipping's will was witnessed by Charles Gilbert, Thomas Gilbert and John Pankhurst.^40^ Its first page clearly refers to ‘real estate in the county of Kent which estate I have lately agreed to sell to my nephew William Kipping’. This land probably relates to Kipping's corner, an area close to Tunbridge in Kent.

It is of note because certain sources note that another Henry Kipping was buried in 1784 at St Mary's church, Hadlow (near Tonbridge), Kent. This is likely to be Henry Kipping, surgeon, of Speldhurst, Kent, who died in or around 1784.^41^

Page two of the will of Henry Kipping of Brighton also refers to a daughter with mental health problems:

by my said wife. And also serving[?] her life to maintain and provide for my daughter Anna who is now unhappily disordered in her mind^40^

A further reference within the will (page two) reveals a philanthropic bequest:

To the said Nathaniel Blaker his executors or administrators by four equal quarterly payments unclear year that is to say the feast of the Annunciation of the blessed Virgin Mary Saint John the Baptist Saint Michael the Archangel the birth of our Lord Christ and the first payment thereof to begin and be made on such of the said feasts or days as shall first and next happen after the unclear of my said wife upon trust that let a ^40^

A codicil to the will was witnessed in 1785 by [?W]Tooke, Charles Gilbert and J Whichelo.^40^ The will was proved on the seventh of November 1785.

## The Kipping family and their stone monument

Records in the Keep archive, Falmer, show many Kippings in Sussex in seventeenth century, including Brighton. This suggests that Kipping may have been from Brighton or the local area. The biography of the Kipping family is recorded in stone inside St Nicholas’ church, Brighton. This wall monument states:

Henry Kipping, Snr, d. 8 Sep 1785, aet 59; his [first] wife, Elizabeth Kipping, d. 1 Dec 1756, aet 26; his [second] wife, Susannah Kipping, d. 15 Apr 1760, aet 26; their issue – John, d. 24 Sep 1760, infant; his [third] wife Anna, d. 12 Apr 1814, aet 74; their issue – John, d. 4 Mar 1774, aet 5; Anna, d. 26 Apr 1805, aet 35; Katherine, d. 11 Aug 1793, aet 18; Henry, d. 25 Mar 1803, aet 25; and William, d. 5 Apr 1855, aet 72; William's wife, Elizabeth, d. 20 Feb 1844, aet 53; their issue - William Henry Kipping, d. 3 Apr 1823, aet 15; and Thomas, d. 1 Dec 1869, aet 60; Thomas’ wife, Mary Ann, d. 14 Dec 1867^42^

## Wider family of Mr Kipping

A later account hints at Mr Kipping's love of country activities and that this was continued by his wider family:

A descendant of Dr Kipping, the late Captain Kipping, of Vernon-terrace, emulated his sporting proclivities; his sad sudden death after a day's hunting [1789] will doubtless be fresh in the remembrance of many, and also his funeral, when his dogs followed the mournful procession to the grave^9^

## Discussion

This paper was commenced in early 2022 and expands upon information in a website biography of Kipping published in January 2023.^19^ Kipping's biography illuminates medical life in Brighton in the second half of eighteenth century, a period associated with more famous names such as Richard Russell and Sir Lucas Pepys. As such, it extends the existing medical biography of Brighton over the past three centuries.^43–48^

Two overarching descriptions of Kipping offer insight into his personality. In the eighteenth century, Fanny Burney described him as ‘curious’ and a ‘prating [sic] good humoured, old gossip, who runs on in as incoherent and unconnected a style of discourse’. The Oxford English dictionary defines the verb ‘prate’ as to ‘talk foolishly or at tedious length about something’. Thus, Burney's use of ‘prating’ appears to reiterate the second part of the sentence. In contrast, Mary Hyde (1977) reached the following description of Kipping: A ‘respected practitioner, good-humored and sympathetic’^26^ That Kipping was a sociable individual – for example fraternising with neighbours and nobility – is clear. The accounts of Kipping's life suggest that contemporary apothecaries did not keep separate personal and professional lives. This is most evident in the duel fought on the very street where Kipping practised. Such a way of life contrasts with the behaviour of later doctors who tended to set their lives aside from wider society. Kipping, of course, lived before the advent of national medical regulation, for example the General Medical Council (1858). It is of note that Kipping's social activities did not seem to prevent him from fulfilling his clinical ‘duties’ or to undermine his reputation.

Kipping was an eighteenth-century practitioner who offered treatments such as bloodletting and unspecified resuscitation techniques. The absence of detail about resuscitation is intriguing but could hint at knowledge of anatomy and circulation. It may be Kipping's medical understanding and approach to treatment earned him the description ‘a worthy disciple of Galen’, i.e., that his care was based upon ancient principles and surpassed in his own life by more modern ideas. His cases offer a glimpse into the challenges of diagnosis, referral and treatment in the eighteenth century. The account of Kipping deferring to Sir Lucas Pepys (in the case of Ralph Thrale) highlights that the practice of an apothecary-surgeon was considered inferior to that of a physician (and, presumably, less well paid too). Kipping appears to have started out as an apothecary (and, by deduction, shopkeeper) and later identified more prominently as a surgeon. This transformation is well described elsewhere:

the average provincial apothecary in 1730 was essentially a shopkeeper who at times practised medicine. The next seventy years were to witness a reversal of these roles to such an extent that by 1800 provincial apothecaries had largely abandoned the drug trade altogether and were engaged in almost full-time practice of medicine^49^

This extension of the clinical role of the apothecary at this time was partly in response to rising demand for medical services and pressure from chemists and druggists who were increasingly selling medicaments and, thus, undercutting apothecaries.^49^ Kipping's transformation from apothecary to surgeon also reflects the growing status of surgeons in England at that time. Evidence for this on a national level lies in the separation of the company of surgeons from the barbers in 1745 and the advances in scientific surgery of pioneers such as John Hunter (1728–1793). The rising status of the London surgeon was enshrined in 1800 when their college received the mark of royal favour.

No evidence suggests that Kipping used water therapy, either mineral or seawater. This may be because Kipping's treatment regimes predate the arrival in Brighton of Richard Russell and his ideas about the benefits of seawater. Thereafter, such treatment could have put him in contention with local physicians, not least Russell. It also suggests that seawater therapy was perceived as a specialised service for qualified physicians and, therefore, Kipping likely made referrals to Russell for the treatment.

Kipping comes across as a colourful character. As a practitioner he was not backward in coming forward to offer medical assistance at a time of urgent need. He was also keen to share his clinical views and experience with others, to the benefit of promoting medical knowledge. By deferring the case of Ralph Thrale to Pepys, Kipping appears to be exercising professional judgement as well as practising within ‘one's area of competence’. An alternative explanation, however, is that Kipping was skilled at recognising and navigating a way around complex clinical problems that were unlikely to reach a favourable outcome.

Kipping appears to have been affluent (owner of a townhouse with servants and silverware) who treated and spent time among members of Brighton's social elite. In contrast to a number of later Brighton doctors,^13^ there is no evidence to suggest that Kipping received an appointment for services to the King's household at Brighton. Kipping was clearly someone passionate about rural pursuits such as horse racing, hunting and dogs. These align with the status of Brighton at the time as a small seaside town with easy access to the Sussex downs (hills). Given Kipping's expertise in swordsmanship it is tempting to deduce a military training, perhaps in a cavalry regiment. Nevertheless, Kipping is not listed in Drew's comprehensive register of commissioned medical officers in the British army.^50^ It remains possible, of course, that he served in a non-medical capacity. Regrettably, more accounts of Kipping and his life do not survive. It is hoped, however, that this biography goes some way towards preserving his memory.
